# Whole-Genome Sequencing of Salmonella enterica Serovar Infantis and Kentucky Isolates Obtained from Layer Poultry Farms in Ecuador

**DOI:** 10.1128/MRA.00091-20

**Published:** 2020-03-26

**Authors:** William Calero-Cáceres, Joyce Villacís, Maria Ishida, Elton Burnett, Christian Vinueza-Burgos

**Affiliations:** a UTA-RAM-One Health, Centro de Investigaciones Agropecuarias, Facultad de Ciencias Agropecuarias, Universidad Técnica de Ambato (UTA), Ambato, Ecuador; b Faculty of Science and Engineering in Food and Biotechnology, Universidad Técnica de Ambato (UTA), Ambato, Ecuador; c New York State Department of Agriculture and Markets, Food Laboratory, Albany, New York, USA; d Institute of Parasitology, McGill University, Montreal, Quebec, Canada; e Facultad de Medicina Veterinaria y Zootecnia, Universidad Central del Ecuador, Quito, Ecuador; University of Maryland School of Medicine

## Abstract

Five strains of Salmonella enterica subsp. *enterica* serovar Infantis and two strains of S. enterica subsp. *enterica* serovar Kentucky isolated in 2017 from Ecuadorian layer poultry farms were sequenced using Illumina MiSeq technology. These isolates were collected on layer farms in central Ecuador, one of the most important areas of egg production in the country. The genome sequences of these isolates show valuable information for surveillance purposes.

## ANNOUNCEMENT

Nontyphoidal *Salmonella* (NTS) represents one of the leading causes of foodborne gastroenteritis worldwide (1, 2). The global burden of NTS infections is 93.8 million cases, and it is estimated that NTS causes around 155,000 deaths annually (3). Unfortunately, there is a lack of information about surveillance studies of NTS in Latin American countries, and reports on NTS outbreaks are scarce in this region (4). In South American countries, there has been an increase in reports of multidrug-resistant Salmonella enterica subsp. *enterica* serovar Infantis in clinical, food, and livestock-related samples. That is the case in Peru (5, 6), Brazil (7), and Ecuador, where reports of NTS infections are related to extended-spectrum β-lactamase (ESBL)-producing *S.* Infantis (8). In Ecuador, this serovar represents the most commonly reported serovar in poultry-related samples (9, 10).

The importance of *S*. Infantis in the epidemiology of nontyphoidal salmonellosis has been described lately in Europe and the United States (2, 11). In the United States, infections caused by CTX-M-65 *S.* Infantis have been linked to travelers to South America (Peru and Ecuador) (12), suggesting that this serotype could play an essential role in the epidemiology of human salmonellosis. No reports of Salmonella enterica subsp. *enterica* serovar Kentucky have been published in Ecuador so far (according to the Scopus and Web of Science databases). In this report, we present the genome sequences of five recent strains of *S.* Infantis and two strains of *S.* Kentucky isolated from layer poultry farms in central Ecuador.

From August 2017 to December 2017, an aleatory sampling program in layer farms was carried out as part of routine diagnostics. Briefly, 102 samples from 21 farms in central Ecuador were analyzed by the ISO-6579 method. The samples were preenriched using buffered peptone water (Merck Millipore, Darmstadt, Germany) and incubated at 37°C for 18 to 24 h. Subsequently, the preenrichments were selectively enriched using Rappaport-Vassiliadis broth (Becton, Dickinson, Le Pont de Claix, France) at 41.5°C for 24 h. Next, the selective enrichment was streaked onto xylose lysine deoxycholate (XLD) agar (Merck Millipore) and incubated at 37°C for 24 h. The presumptive colonies were purified in MacConkey agar (Merck Millipore) and incubated at 37°C for 24 h. Biochemical tests were performed to confirm the genus *Salmonella* (catalase, oxidase, triple sugar iron [TSI], Simmons citrate, Christensen urea agar base, indole reaction). The positive samples were cryopreserved using overnight growth in LB broth (Sigma-Aldrich, St. Louis, USA) supplemented with 30% glycerol (Merck Millipore).

Thirty-one isolates were identified as belonging to the genus *Salmonella*. Seven isolates were selected according to their sampling origin (strains U822s, U824s, and U842s, Latacunga; U825s, Quero; U845s, Ambato; U827s, Pelileo; and U828s, Cevallos). Isolates were recovered from poultry feeders, environmental swabs, and cloacal swabs (Table 1). *Salmonella* cultures were grown in LB broth and incubated at 37°C for 24 h. The biomass was used for genomic DNA extraction using the Wizard genomic DNA purification kit (Promega, Madison, WI, USA) according to the manufacturer’s instructions and DNA quantified using the Qubit 3.0 fluorometer (Invitrogen, CA, USA). Genomic libraries were prepared using the Nextera XT library prep kit (Illumina, CA, USA), and whole-genome sequencing (WGS) was performed using 300-bp paired-end read lengths on the MiSeq platform (Illumina). Raw reads were *de novo* assembled via SKESA v2.2 using the NCBI Prokaryotic Genome Annotation Pipeline (13). The draft genome assembly quality was evaluated using CheckM v1.0.18 (14) and QUAST v4.4 (15).

**TABLE 1:** Whole-genome sequencing-based characterization of 7 nontyphoidal Salmonella enterica strains isolated from layer poultry farms in Ecuador

Laboratory identifier	Sample type	Predicted S. enterica subsp. *enterica* serovar	No. of reads	No. of contigs	Total length (bp)	*N*_50_ (bp)	GC content(%)	No. of genes	CheckM quality (% completeness [% contamination])	Genome coverage (×)	MLST	Plasmid replicon	Pathogenicity islands	GenBank accession no.	SRA accession no.
U822s	Feed	Infantis	923,027	279	4,970,844	34,389	52.37	5,087	100 (0.08)	71	32		SPI-13, SPI-14, SPI-5	AACYLG000000000	SRR8266241
U824s	Feed	Infantis	663,871	322	4,926,207	29,447	52.35	5,051	100 (0.08)	52	32		SPI-13, SPI-14, SPI-5, SPI-1, C63PI	AADKME000000000	SRR8282892
U825s	Feed	Infantis	1,173,443	220	4,990,278	47,232	52.29	5,100	100 (0.08)	103	32		SPI-13, SPI-14, SPI-5, SPI-1, C63PI	AADPVC000000000	SRR8282855
U827s	Environmental swabs	Kentucky	1,308,655	285	4,744,299	40,935	52.18	4,793	100 (0.39)	99	152	IncX1	SPI-8, C63PI	AADPVF000000000	SRR8282852
U828s	Environmental swabs	Kentucky	926,830	192	4,751,148	52,705	52.15	4,788	100 (0.39)	83	152	IncX1	SPI-8, C63PI	AADFDT000000000	SRR8282866
U842s	Environmental swabs	Infantis	1,163,477	179	5,001,560	52,646	52.22	5,089	100 (0.08)	83	32		SPI-13, SPI-14, C63PI, SPI-5	AACYLE000000000	SRR8266239
U845s	Cloacal swabs	Infantis	760,589	298	4,948,982	30,021	52.34	5,064	100 (0.08)	62	32		SPI-13, SPI-14, C63PI, SPI-3, SPI-5	AADKMC000000000	SRR8282870

Draft genomes were analyzed using the tools from the Center for Genomic Epidemiology of the Technical University of Denmark. Using WGS data, the serotypes were determined using SeqSero v1.2 (16). Multilocus sequence types (MLSTs) were determined using MLST v2.0 (17). The presence of plasmids was identified using PlasmidFinder v2.1 (18). The prediction of bacterial pathogenicity was evaluated using PathogenFinder v1.1 (19). Finally, the detection of *Salmonella* pathogenicity islands was carried out using SPIFinder v1.0 (20). All analyses were performed using default parameters.

The number of contigs ranged from 179 to 322, and their *N*_50_ values ranged from 29,447 to 52,705 bp. The draft genomes ranged from 4,744,299 to 5,001,560 bp, with 52.27% average GC content (Table 1). The assemblages showed 100% completeness in all isolates, and the percentage of contamination ranged from 0.08% to 0.39%. The number of annotated genes ranged from 4,788 to 5,100. The SeqSero analysis revealed that the isolates belonged to the S. enterica subsp. *enterica* serovars Infantis (5) and Kentucky (2). MLST analysis showed that the *S.* Infantis strains belonged to sequence type 32 (ST32) and the *S.* Kentucky strains to ST152. The plasmid replicon typing detected the plasmid incompatibility group IncX1 in the *S.* Kentucky strains only (Table 1).

These draft genomes provide essential information about the circulating clones of *Salmonella* from layer poultry farms in Ecuador and can serve as references for genome comparison studies.

### Data availability.

The draft genome sequences reported in this article were deposited in GenBank under the accession numbers AACYLG000000000, AADKME000000000, AADPVC000000000, AADPVF000000000, AADFDT000000000, AACYLE000000000, and AADKMC000000000.

## References

[1] World Health Organization. 2015 WHO estimates of the global burden of foodborne diseases. World Health Organization, Geneva, Switzerland.

[2] EFSA, ECDC. 2018 The European Union summary report on trends and sources of zoonoses, zoonotic agents and food-borne outbreaks in 2017. EFSA J 16:e05500. doi:10.2903/j.efsa.2018.5500. PMC7009540 32625785

[3] Majowicz SE, Musto J, Scallan E, Angulo FJ, Kirk M, O'Brien SJ, Jones TF, Fazil A, Hoekstra RM, International Collaboration on Enteric Disease “Burden of Illness” Studies 2010 The global burden of nontyphoidal *Salmonella* gastroenteritis. Clin Infect Dis 50:882–889. doi:10.1086/650733. 20158401

[4] Balasubramanian R, Im J, Lee JS, Jeon HJ, Mogeni OD, Kim JH, Rakotozandrindrainy R, Baker S, Marks F 2019 The global burden and epidemiology of invasive non-typhoidal *Salmonella* infections. Hum Vaccin Immunother 15:1421–1426. doi:10.1080/21645515.2018.1504717. 30081708 PMC6663144

[5] Granda A, Riveros M, Martínez-Puchol S, Ocampo K, Laureano-Adame L, Corujo A, Reyes I, Ruiz J, Ochoa TJ 2019 Presence of extended-spectrum β-lactamase, CTX-M-65 in *Salmonella enterica* serovar Infantis isolated from children with diarrhea in Lima, Peru. J Pediatr Infect Dis 14:194–200. doi:10.1055/s-0039-1685502.

[6] Silva C, Betancor L, García C, Astocondor L, Hinostroza N, Bisio J, Rivera J, Perezgasga L, Pérez Escanda V, Yim L, Jacobs J, García-del Portillo F, SalmoIber CYTED Network, Chabalgoity JA, Puente JL 2017 Characterization of *Salmonella enterica* isolates causing bacteremia in Lima, Peru, using multiple typing methods. PLoS One 12:e0189946. doi:10.1371/journal.pone.0189946. 29267322 PMC5739443

[7] Monte DF, Lincopan N, Berman H, Cerdeira L, Keelara S, Thakur S, Fedorka-Cray PJ, Landgraf M 2019 Genomic features of high-priority *Salmonella enterica* serovars circulating in the food production chain, Brazil, 2000–2016. Sci Rep 9:1–12. doi:10.1038/s41598-019-45838-0. 31363103 PMC6667439

[8] Cartelle Gestal M, Zurita J, Paz y Mino A, Ortega-Paredes D, Alcocer I 2016 Characterization of a small outbreak of *Salmonella enterica* serovar Infantis that harbor CTX-M-65 in Ecuador. Braz J Infect Dis 20:406–407. doi:10.1016/j.bjid.2016.03.007. 27215783 PMC9427545

[9] Salazar GA, Guerrero-López R, Lalaleo L, Avilés-Esquivel D, Vinueza-Burgos C, Calero-Cáceres W 2019 Presence and diversity of *Salmonella* isolated from layer farms in central Ecuador. F1000Res 8:235. doi:10.12688/f1000research.18233.2. 31069068 PMC6480948

[10] Vinueza-Burgos C, Cevallos M, Ron-Garrido L, Bertrand S, De Zutter L 2016 Prevalence and diversity of *Salmonella* serotypes in Ecuadorian broilers at slaughter age. PLoS One 11:e0159567. doi:10.1371/journal.pone.0159567. 27414038 PMC4944992

[11] Tate H, Folster JP, Hsu CH, Chen J, Hoffmann M, Li C, Morales C, Tyson GH, Mukherjee S, Brown AC, Green A, Wilson W, Dessai U, Abbott J, Joseph L, Haro J, Ayers S, McDermott PF, Zhao S 2017 Comparative analysis of extended-spectrum-ß-lactamase CTX-M-65-producing *Salmonella enterica* serovar Infantis isolates from humans, food animals, and retail chickens in the United States. Antimicrob Agents Chemother 61:e00488-17. doi:10.1128/AAC.00488-17. 28483962 PMC5487606

[12] Brown AC, Chen JC, Watkins LKF, Campbell D, Folster JP, Tate H, Wasilenko J, Van Tubbergen C, Friedman CR 2018 CTX-M-65 extended-spectrum β-lactamase–producing *Salmonella enterica* serotype Infantis, United States. Emerg Infect Dis 24:2284–2291. doi:10.3201/eid2412.180500. 30457533 PMC6256390

[13] Souvorov A, Agarwala R, Lipman DJ 2018 SKESA: strategic k-mer extension for scrupulous assemblies. Genome Biol 19:153. doi:10.1186/s13059-018-1540-z. 30286803 PMC6172800

[14] Parks DH, Imelfort M, Skennerton CT, Hugenholtz P, Tyson GW 2015 CheckM: assessing the quality of microbial genomes recovered from isolates, single cells, and metagenomes. Genome Res 25:1043–1055. doi:10.1101/gr.186072.114. 25977477 PMC4484387

[15] Gurevich A, Saveliev V, Vyahhi N, Tesler G 2013 QUAST: quality assessment tool for genome assemblies. Bioinformatics 29:1072–1075. doi:10.1093/bioinformatics/btt086. 23422339 PMC3624806

[16] Zhang S, Yin Y, Jones MB, Zhang Z, Deatherage Kaiser BL, Dinsmore BA, Fitzgerald C, Fields PI, Deng X 2015 *Salmonella* serotype determination utilizing high-throughput genome sequencing data. J Clin Microbiol 53:1685–1692. doi:10.1128/JCM.00323-15. 25762776 PMC4400759

[17] Larsen MV, Cosentino S, Rasmussen S, Friis C, Hasman H, Marvig RL, Jelsbak L, Sicheritz-Pontén T, Ussery DW, Aarestrup FM, Lund O 2012 Multilocus sequence typing of total-genome-sequenced bacteria. J Clin Microbiol 50:1355–1361. doi:10.1128/JCM.06094-11. 22238442 PMC3318499

[18] Carattoli A, Zankari E, Garciá-Fernández A, Larsen MV, Lund O, Villa L, Aarestrup FM, Hasman H 2014 In silico detection and typing of plasmids using PlasmidFinder and plasmid multilocus sequence typing. Antimicrob Agents Chemother 58:3895–3903. doi:10.1128/AAC.02412-14. 24777092 PMC4068535

[19] Cosentino S, Voldby Larsen M, Møller Aarestrup F, Lund O 2013 PathogenFinder—distinguishing friend from foe using bacterial whole genome sequence data. PLoS One 8:e77302. doi:10.1371/journal.pone.0077302. 24204795 PMC3810466

[20] Center for Genomic Epidemiology. 2020 SPIFinder (version 1.0). https://cge.cbs.dtu.dk/services/SPIFinder/.

